# Improving Treatment of Elderly Patients by Interprofessional Education in a Quality Network of Geriatric Medicine: Protocol for Evaluating an Educational Initiative

**DOI:** 10.2196/11067

**Published:** 2019-05-03

**Authors:** Daisy Huenefeld, Sibyll Rodde, Gertrud Bureick, Barbara Elkeles, Joachim Hasebrook

**Affiliations:** 1 Board of the Foundation St Franziskus Foundation Muenster Germany; 2 zeb.healthcare zeb.rolfes.schierenbeck.associates Muenster Germany; 3 BIGi Project St Franziskus Foundation Muenster Germany; 4 Klinik Maria Frieden Telgte Germany; 5 zeb.business school Steinbeis University Berlin Muenster Germany

**Keywords:** geriatrics, education, medical, continuing, education, nursing, continuing, staff development, clinical competence, interdisciplinary placement

## Abstract

**Background:**

All statistics on the development of demand for care for multimorbid elderly patients highlight the acute pressure to act to adequately respond to the expected increase in geriatric patient population in the next 15 years. Against this background, great importance must be attached to the improvement of cross-occupational group and cross-sector treatment of these patients. In addition, many professionals in the health care sector often have little knowledge about the special treatment and care needs of the elderly.

**Objective:**

The Quality Network of Geriatric Medicine in north-west Germany is the body responsible for the project; with its member organizations, it provides care for over 400,000 inpatients and is thus one of the largest associations for geriatrics in Germany. The Quality Network conducts binding evaluated qualification measures for staff involved in the treatment and care of multimorbid elderly patients. The training offers are especially intended for staff who have not yet been trained in working with elderly patients. This approach is intended to improve the expertise of various occupational groups on different hierarchy levels, to include patients and their family members in the evaluation process, and to initiate changes within the organizations.

**Methods:**

Various instruments are used in the evaluation of qualification measures: besides written surveys and questionnaires, structured work groups (consensus groups) and interviews are conducted. The evaluation starts before the qualification measures to determine the starting point and then continues during the measure and after its completion. This allows major findings to be integrated directly into the ongoing qualification program. At least 100 trainings on geriatric topics, 80 consensus groups, and 120 patients (and family members) are going to be included in the study.

**Results:**

The evaluation of the educational initiative is funded by the State of Northrhine-Westfalia (Germany; LZG TG 71 001 / 2015 and LZG TG 71 002 / 2015). The results of the study will be published after review and approval by the state authorities – presumably by the end of 2019. The before and after comparison of the treatment-related outcomes at the beginning and near the completion of the educational initiative gives insights into how transfer-oriented education can improve the treatment of elderly patients across sector lines for inpatients as well as outpatients. The evaluation of the implementation of educational content in day-to-day work and occupational groups is to facilitate recommendations about economically sensible use of educational resources and about further adjustments to the training content.

**Conclusions:**

The evaluation develops the foundation for targeted and needs-oriented qualification measures as well as transfer in cross-sector, multiprofessional networks. Instruments and results will be published and provided to other health care networks and institutions. The Quality Network will implement the results of the evaluation process in its member institutions.

**International Registered Report Identifier (IRRID):**

DERR1-10.2196/11067

## Introduction

The average proportion of people older than 65 years in the Organisation for Economic Co-operation and Development countries increased from 9% in 1969 to 15% in 2010 and by the year 2050 will have reached approximately 27% [1]; at present, one-fifth (20.7%) of the German population is older than 65 years [2]. Already in the year 2020, which is approaching fast, 2 out of every 3 hospital beds will be occupied by patients older than 60 years—an age group that often has at least one chronic disease [3,4]. For medical care, this means that the symptoms that the admission diagnosis relates to should not be treated in isolation but that other preexisting diseases of patients also need to be treated appropriately [5]. This increasingly leads to problems in the medical care for elderly patients [6]. In addition, risks that arise from falls, malnutrition, and polypharmacy are often not given sufficient attention. Consequences can include poor treatment results with (partially) restricted quality of life or even mortality [5,6].

Globally, the next 20 years will see a duplication of the prevalence of dementia-type illnesses [7]. Especially the very old often suffer from cognitive impairments or dementia, which also increases the vulnerability of this patient group. This is however often not known upon admission and can have an adverse effect on the (course of) treatment. The specific vulnerability of elderly people demands specialized expert treatment.

### Need for Qualification

The quality of elderly medical care depends to a major degree on the quality and quantity of motivated, well-trained professionals [8]. Three-fourths of the more than 1800 ward and department heads surveyed throughout Germany responded that they regard training of their staff in dementia topics as necessary [9]. Other studies show that the specialist diagnostic knowledge of physicians to distinguish between dementia and delirium often does not meet the necessary requirements [10].

Organizational processes in hospitals need to match the needs of this vulnerable patient group, especially patients suffering from dementia [11]. Therefore, the geriatric knowledge of all parties who come into contact with elderly people (physicians, nursing staff, therapists, social workers, and administration) needs to be improved [12].

The improvement of communicative conditions and skills of all care providers leads to a greater quality of care results, improved patient satisfaction, and higher work satisfaction of staff [13]. Furthermore, the exchange between colleagues can contribute to the positive development of collaboration across sector borders. In this way, it would be possible to prepare and define the postinpatient care environment already during an inpatient stay [14].

Qualification measures for the involved occupational groups are generally offered on a voluntary basis and often used by interested staff or organizations with a geriatric focus. However, institutions that would benefit the most from trainings in geriatric topics often do not take part. There is thus a risk that educational measures will not reach those care providers and institutions that have not yet recognized the importance of the demographic change [15].

Experiences with a work shadowing program at the St. Franziskus Hospital in Münster show that exclusively training 1 occupational group is insufficient because real changes to the care situation can only be achieved through interaction between different occupations [16].

The Qualitätsverbund Geriatrie Nord-West-Deutschland (in English: Quality Network of Geriatric Medicine in north-west Germany) is a group of more than 65 inpatient and outpatient institutions including hospitals with and without geriatric departments, inpatient geriatric rehabilitation facilities, elderly care institutions, outpatient care services, networks of doctor’s offices, and outpatient rehabilitation providers.

### Objectives

The main objective of this project is improved care for elderly people in different inpatient and outpatient institutions of the Quality Network. The project involves systematically evaluating knowledge transfer and learnings. In addition, necessary structural changes, for example in work procedures, for improvement of the care situation for elderly patients are to be introduced and established for the near future. This project is a study to evaluate geriatric training measures and thus not a clinical trial. The project is subsidized by the Landeszentrum Gesundheit Nordrhein-Westfalen (North Rhine-Westphalian Center for Health). Participating institutions incur no costs for the project.

The guiding research questions are as follows:

- Do all parties who come into contact with elderly people attend courses for geriatric topics? Are the participants satisfied with the content of the training? Do they suggest improvements?
- Is the transfer of expertise in their everyday working environments successful? Do the participants recommend that others take part in the training session?
- How many consensus groups involving people from different occupations in the institutions work toward targeted measures for improved care of elderly patients in their place of work? Is the implementation of measures/actions effective?
- Do patients notice any positive change in view of the treatment at the end of the project?
- Do physicians and caregivers apply geriatric screenings and assessments more often at the end of the project?

## Methods

### Subjects

On the network’s initiative, the level and need for education of the staff were analyzed in 26 institutions before the actual start of the project. Data are available for 2200 people.

**Figure 1:**
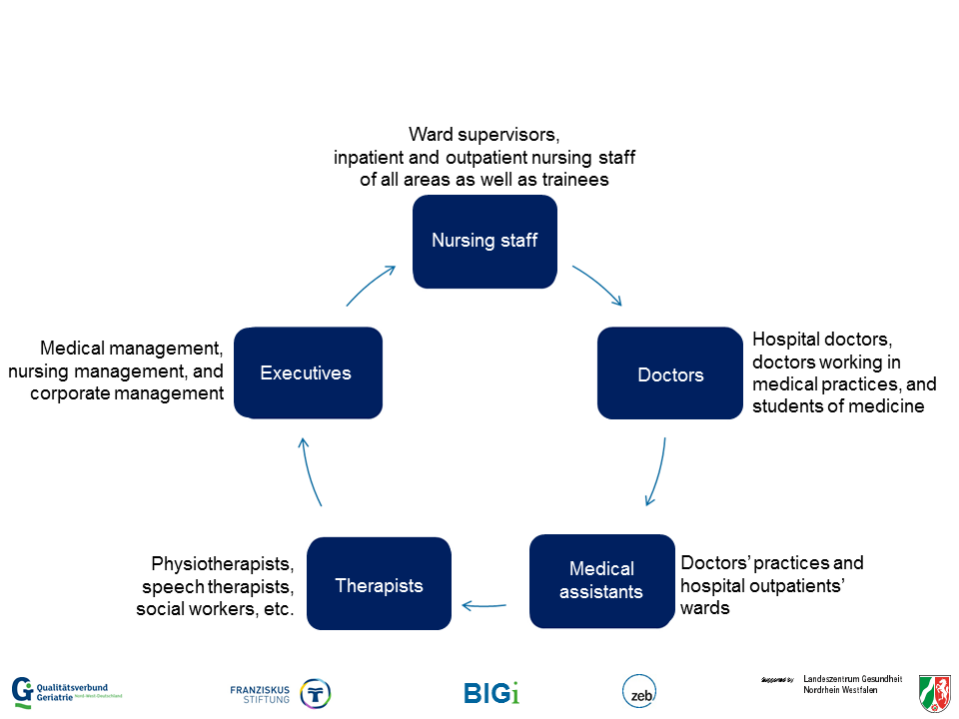
Overview of occupational groups involved in the educational initiative.

At least 1000 people who took part in a total of 100 trainings on geriatric topics were included in the recent education evaluation. This sample includes people from all hierarchy levels and occupational groups who come into contact with elderly patients (see Figure 1). In addition, 125 consensus group meetings (with approximately 4-8 participants) are to be evaluated so far so that approximately. 500 to 1000 more people are involved in this evaluation step.

Participation in the study was voluntary. All participants were informed in writing about the aims and process of the study before anonymous data collection. Participants agreed with a second survey 6 months later (paper or Web-based), and for this reason, many provided their email address to the study leaders. The Web-based questionnaire was presented by a professional tool that guaranteed data protection, data security, and anonymity. As the study’s purpose was to evaluate training measures, the prior permission from an ethics commission was not required.

The patient sample includes people who are 75 years old or older and who are being treated for 3 days or longer as an inpatient for accident or abdominal surgery. At the beginning and near the end of the data collection in the project, patients and their family members are surveyed about inpatient treatment and the quality of life and health. Patients and their families are informed in writing and also told about the aims of the project and provide their written permission to take part in the survey. Participation is voluntary. There are plans to survey 60 patients and family members at the start (as a prestudy) and another 60 patients and family members at the end (poststudy) of data collection. In total, approximately 1800 people are included in the project.

### Design

The educational initiative aims at all staff members of the institutions who are included in the care and treatment of elderly, multimorbid patients. This includes nursing staff, physicians, therapists, auxiliary staff, medical assistants, social workers, medical students, trainees, as well as people from administration and management.

The educational measures are evaluated at various measuring times (before and after) and with regard to various contents and target groups.

As the *baseline*, the level and need for education of staff members regarding geriatric topics are assessed. All occupational groups that have contact with elderly patients (see Figure 1) are included in the survey. This should identify the training topics that are particularly important or missing from the training offer. This assessment took place before the actual project start on the Quality Network’s own initiative. In total, 26 institutions took part with more than 8200 respondents.

The evaluation mainly comprises existing training offers about 20 diverse subject areas with a geriatric focus. The educational measures are on topics such as *Dealing with patients with dementia*, *Using geriatric screenings and assessments*, or *Nutrition for the elderly.* Depending on the topic, the trainings are intended for caregivers, physicians, other health professionals, and various management staff. The questionnaires for the evaluation were developed at the start of the project, together with academic experts, based on the standards for transfer-oriented education [17]. *Directly before* and *directly after* geriatric trainings, the participants are asked about their expectations and their satisfaction regarding the course. The regular feedback from course participants should indicate whether prior knowledge and experiences are considered to adapt the training offer according to needs in the medium term. *After 6 months,* the participants are asked how well they have transferred the training content into their day-to-day work. Both directly after the course and 6 months later, the participants specify whether they would recommend that others take part in the training session. Ideas from the trainings should be specifically implemented in the network institutions and adapted to the specific requirements in inpatient and outpatient settings. Work groups/quality groups involving people from different occupations in the institutions and using the nominal group technique [18] work toward targeted measures for improved care of elderly patients at their place of work. The consensus groups are evaluated *alongside* their activities and *3 months after completion*. To do so, meeting minutes, questionnaires, and interview data are evaluated qualitatively and quantitatively [8,19].

To determine a patient-related outcome, assessments from patients and family members/caregivers as well as various aspects of the treatment are gathered. *At the start* and *near the end of the data collection*, patients and family members assess the treatment. In this context, it is recorded whether and which specific geriatric screenings and/or assessments were conducted during the treatment. A meta-analysis about comprehensive geriatric assessments showed that use of assessments led to improved patient-related outcomes as well as reduced costs [20]. Moreover, applying assessments helps to identify vulnerable patients and adapt therapies to their specific needs better than without assessments [21,22].

**Figure 2:**
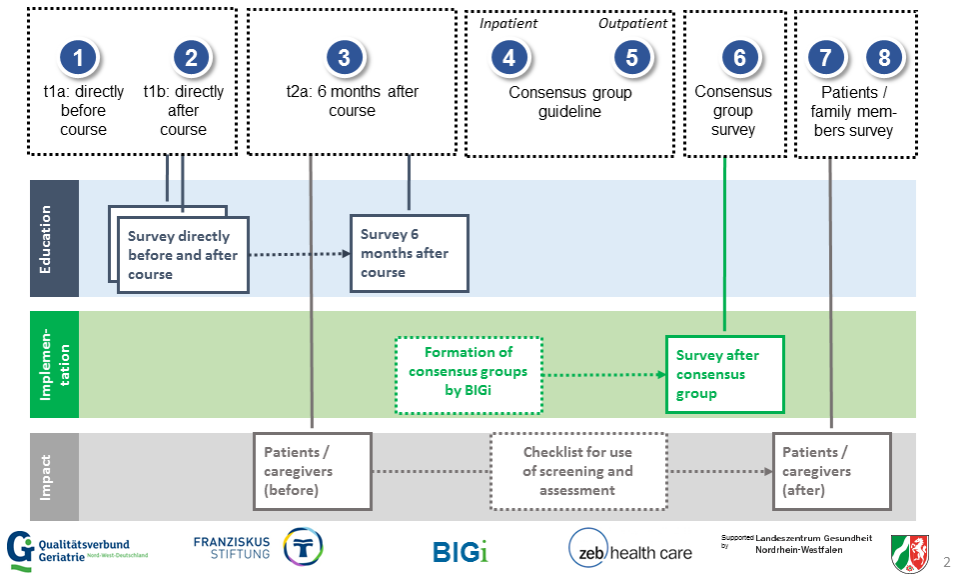
Overview of measuring times and evaluation instruments.

The questionnaire for patients includes the items for the German language Short Form (SF)-12 for recording health-related living quality [23,24], and it also includes items relating to the course of treatment. The content of the family member questionnaire corresponds to the patients’ questionnaire. The items of each questionnaire were developed together with clinical experts, with the exception of the SF-12 items. An overview of the measuring times and evaluation instruments is depicted in Figure 2.

### Material

#### Gathering Data on Level and Need for Education

The questionnaire used before project start for recording the level of education and need for education about geriatric topics was divided into 4 chapters, each dealing with 1 theme. The first chapter included 3 items. The staff members specified whether further training about geriatric topics is offered in their organization and whether they themselves have taken part in internal or external geriatric trainings. The questions could be answered with “Yes,” “No,” or “No response.” The second chapter included a list of 28 geriatric training topics such as “Communicating with patients who have dementia” or “Promoting mobility.” The staff members judged their needs for training about these topics (possible answers were “Yes,” “No,” or “No response”) and selected any trainings that they had already taken part in. The third chapter focused on assessing prior trainings. In total, 19 items related to aspects of preparation and conducting of prior trainings as well as the implementation of content into day-to-day work. Respondents could choose from “Applies in all cases,” “Applies partially,” “Hardly applies,” “Does not apply,” and “No response.” The final question was based on an assessment of the likelihood of recommendation and was phrased as follows: “I recommend participating in trainings on geriatric topics to my colleague.” The possible answers were the same as above. The last part of the survey was for demographic details.

#### Evaluation of Geriatric Trainings

Findings from economic education research point out that success factors for educational measures are to be recorded using a phase-oriented perspective (preparation of the measure, conduct, transfer of learnings; [25,26]). This phase-oriented approach was considered in the development of the questionnaires for evaluating geriatric trainings.

The questionnaire filled out by participants *immediately before* the training includes 12 statements that can be answered with the following alternative responses: “Applies in all cases,” “Applies partially,” “Hardly applies,” “Does not apply,” and “No response.” The first 2 items relate to the motivation to take part in the course (personal interest and recommendation from employer). The remaining statements mention options for personal preparation and expectations in the course and regarding implementation of the content into day-to-day work. For example, the items include the following: “I have received a lot of information about the contents of this course in advance,” “It is important to me that this course provides sufficient time to consolidate and practice,” and “It is important to me that there are sufficient opportunities after this course to put the learnings into practice.” Respondents can also note their personal wishes regarding the respective courses in 3 free textboxes. The questions are as follows: “What is particularly important to you in this course?,” “Future courses must...,” and “In future courses, I do not want to....” Finally, demographic data are collected.

The survey filled out *immediately after* the training includes 10 statements with the possible responses: “Applies in all cases,” “Applies partially,” “Hardly applies,” “Does not apply,” and “No response.” The items relate to the assessment of training content, the behavior of the trainer, and the support of the employer. They are phrased as follows: “The contents of the course matched completely with my needs in day-to-day work,” “The course trainer recognized problems and needs of participants and addressed them in the course,” and “My organization ensured my smooth participation in the course.” The tenth statement measures the likelihood of recommendation and is phrased as follows: “All in all, I would recommend this training to a colleague.” Here also, 3 free text boxes are provided for participants to note their positive and negative impressions about the training as well as recommendations for improvement. Finally, demographic details are requested.

The survey on *implementing training content into day-to-day work* is sent *6 months after the end of the course* as either a paper or Web-based version. The 6 statements have the aforementioned answer categories and relate to the practical use of the training content and the perceived support and appreciation from colleagues and/or line managers in implementing the training content into day-to-day work. The items include, for example, “The contents of the training help me in practical dealings with elderly patients” and “My knowledge from the training and my efforts in the implementation of improvement measures were appreciated in my organization.” The final statement is to measure the likelihood of recommendation. In 2 free textboxes, the respondents can note whether and which training content they can implement in their day-to-day work. The final section is a collection of demographic data.

**Figure 3:**
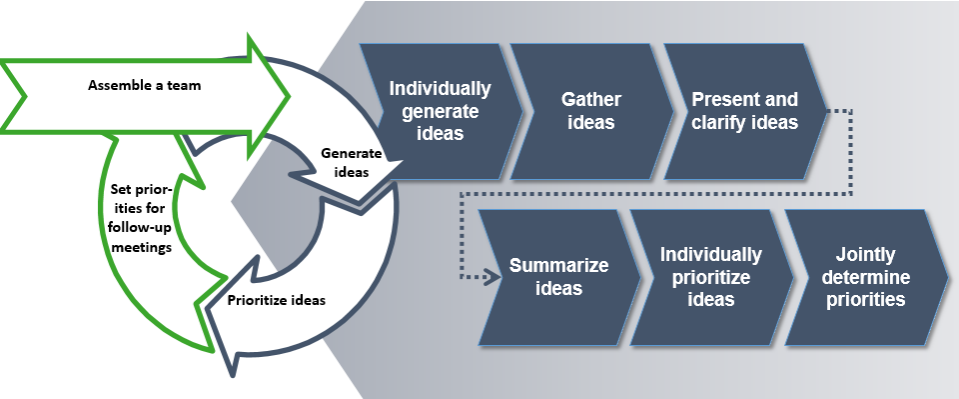
Agenda for a consensus group meeting.

#### Conducting and Evaluating Consensus Groups

A guideline (including information letters and worksheets) for preparing and conducting consensus groups was developed for practical use as part of the project. It serves as a handbook for facilitators who give the meetings organizational and content structure. Appropriate adjustments were made to consider the diverse requirements in inpatient and outpatient settings. The agenda for a consensus group meeting is presented in Figure 3.

Meeting minutes document the characteristics, such as duration, topic, preferred ideas, occupational groups, and gender of the facilitator. At the end of the consensus group meeting, the participants and the facilitator assess the group work in writing based on 4 statements and the possible responses: “Applies in all cases,” “Applies partially,” “Hardly applies,” “Does not apply,” and “No response.” The items are as follows: “Suggestions for improved care for elderly, multimorbid patients were developed and assessed together,” “The cooperation of various occupational groups was helpful for developing improvement suggestions for the benefit of elderly, multimorbid patients,” and “The fact that the meeting(s) were supported by a facilitator was helpful for the content-related work of the group.” The final question is based on the likelihood of recommendation. In 3 free textboxes, participants can note what they found to be helpful or unhelpful for the work in the consensus group and any ideas they might have for improving similar work groups. Then 3 months after the completion of the consensus groups, institution managers are interviewed in a 25-min, structured phone call about whether and to what degree the initiated change suggestions were able to be implemented in the institution.

#### Assessment of the Treatment-Related Outcome

The treatment-related outcome is assessed from the following 2 perspectives: (1) use of geriatric screening and assessment instruments during the course of treatment, and (2) assessment of inpatient treatment by patients and family members.

##### Patient and Family Member Survey

Surveys for patients and family members relate to each other in terms of content and mentioned aspects of inpatient treatment and later discharge, which are particularly relevant for elderly, multimorbid patients. For example, there are questions on whether the patients receive sufficient help in eating and drinking as well as explanation about outpatient support. All items are phrased as questions and adapted to the respective readers, for example, “Were your pre-existing conditions/accompanying conditions considered by the nursing staff during your treatment?” (patient survey) compared with “Were the pre-existing conditions/accompanying conditions of your family member considered by the nursing staff during treatment?” (family member survey). The response categories are “Yes, completely,” “Somewhat,” “No,” “Was not necessary,” and “I don’t know.”

An assessment of the state of health before the current hospital stay is requested in both surveys. The patient survey includes the SF-12 survey as a standardized measurement instrument about the state of health [23]. In addition, demographic data about the patients is gathered.

##### Further Measures Including Frequency of Geriatric Screenings/Assessments

A check list is used to record whether any screenings and assessments, which are recommended for elderly medical care [27], were conducted during inpatient treatment. By using the screening instruments, important diagnostic information for the treatment can be gathered, for example, regarding the general level of functioning, the nutritional state, or the risk of delirium of the patient [28], as these aspects are considered to be of high relevance for an optimal outcome especially after surgery [29,19].

Furthermore, it is recorded whether the social services were involved in the preparations for discharge and/or family members were informed about current medication or nutritional recommendations, as sufficient information seems to have a positive influence in the course of treatment [30]. In addition, demographic patient data are gathered. Within the project, neither individual patient data nor any screening or assessments results have been stored. Rather, it has only been recorded whether screening and assessment instruments have been used and whether the main aspects—mobility, nutrition and cognition—have been covered because screening and assessments have proven to show positive impact on output and outcome of geriatric treatment [20-22].

### Procedure

To prepare for the evaluation, at the start of the project, the following 3 work groups were formed, which included project managers as well as experts from the fields of geriatrics, surgery, general medicine, inpatient and outpatient care, therapy, further training, quality management, and administration:

- The first work group discussed and agreed upon the evaluation tools, such as surveys, and the procedure how to collect data from the geriatric training courses in the participating institutions.
- The second work group addressed the conducting and evaluation of consensus groups/quality groups.
- The third work group focused on recording treatment-related outcomes. The assessment of the treatment by patients and family members was regarded as fundamental. Furthermore, the recording of certain geriatric screenings/assessments as well as discharge management were seen as important.

Data collection began in June 2016 and was completed by the end of 2018. Data analysis and final reporting are currently in progress.

#### Data Collection on Level of Education

On the Quality Network’s initiative, the level and need for education of staff members regarding geriatric topics were assessed in advance. Members of various occupational groups who are involved in the care for multimorbid, elderly patients received a survey about the level and need for education. The results of these surveys were assessed specifically per institution. At the same time, each participating institution received a benchmark report to be able to compare the results of their own organization with those of others. The latest survey results are used in revising existing educational measures and are considered in the development of new ones.

#### Evaluation of Geriatric Trainings

Training participants complete a survey relating to their expectations at the start of the course and another about their level of satisfaction with the course at the end. The surveys are handed out, collected, and submitted to the project manager by the trainer. The education experts at the institutions receive a quantitative and qualitative analysis for the various courses. Then, 6 months after the end of the training, course participants specify to what degree they have been able to implement the education content into their day-to-day work. This survey on the subsequent assessment is sent to the participants as a paper of Web-based version.

#### Conducting and Evaluating Consensus Groups

Generally, 4 to 8 staff members from various occupational groups, fields, and hierarchy levels take part in a consensus group/quality group (up to 4 times for approximately 30-60 min). Relating to a specific question about improved care for elderly patients, ideas for measures should be developed in the group, and the implementation of these measures should be initiated. For conducting and evaluating, various work materials including guidelines have been developed. A facilitator can support the group in finding ideas and developing a common suggestion. Using meeting minutes, specific course characteristics such as the topic of the work meeting and the preferred suggestion should be documented. In a survey at the end of the consensus group, the group work will be assessed, and meeting minutes will be created. Finally, the facilitator will submit the suggestion to the institution’s management and ask for a review and approval. Upon approval, the details about specific measures will be recorded in writing and their implementation into day-to-day work will begin.

Then, 3 months after the completion of the consensus groups/quality groups, representatives of the institutions will be interviewed in a structured interview about whether and to what degree the initiated change suggestions were able to be implemented.

#### Treatment-Related Outcome

The patient sample includes people who are 75 years old or older and who are being treated for 3 days or longer as an inpatient for accident or abdominal surgery. At the beginning and near the end of the data collection in the project, patients and their family members are surveyed about the completed treatment and the quality of life and health. Surveys for patients and family members relate to each other in terms of content and mention aspects of inpatient treatment and later discharge, which are particularly relevant for elderly, multimorbid patients, among other topics. An assessment of the state of health before the current hospital stay is requested in both surveys. The patient survey includes the SF-12 survey as a standardized measurement instrument about the state of health [26].

To assess the outcome, a checklist is used to record whether certain screenings and assessments that are recommended for elderly medical care [26] were conducted during inpatient treatment. By using these instruments, significant diagnostic information for the treatment can be gathered, for example, regarding the general level of functioning, the nutritional state, or the risk of delirium of the patient [27]. Furthermore, it is analyzed whether the social services were involved in the preparations for dismissal and/or family members were informed about current medication. Patient-related information about the courses of treatment is not part of the educational evaluation.

## Results

We expect academically sound conclusions about how the patient-related medical outcome is improved through the transfer of educational and accompanying measures in the intersector care of elderly patients. Furthermore, particularly changes at the Quality Network’s institutions actively involved in education and those who offer and carry out many courses should be compared with the changes of results in less active institutions. We expect that this comparison will provide detailed conclusions about the necessary adjustments to standard procedures, process instructions, organizational guidelines, educational plans, and training guidelines.

From the evaluation of how educational content has been implemented after visiting a course and through the formation of consensus groups/quality groups, we expect conclusions about the economically viable use of educational measures in improving patient care. The before and after comparison at institutions with high transfer rates, that is, specific implementations of learnings through measures that are developed in consensus groups/quality groups, is also expected to provide starting points for optimizing the use of medical resources and preventing cost-driving revolving door effects.

## Discussion

The individual competence of individual members of the treatment team for geriatric patients can be viewed as a source of collective competence development and vice versa. The knowledge to be gained for improving elderly medical care is meant to facilitate finding solution patterns for new tasks and shifting requirements and/or transferring knowledge and competence to the organization, the group, and the Quality Network in cooperation with other experts and thus to promote organizational learning [31].

### First Results

Initial results and experiences clearly show that the offers of the interprofessional educational initiative in geriatrics are met with serious interest from all occupational groups and that the various groups are all taking part. At the same time, the survey results about the level and need for education confirm the results of other studies in this field [9,16]. The survey results also give the responsible people in participating institutions the possibility to address the individual training needs of their staff members. They can then work on ensuring that any “blind spots” in geriatric fields are closed and that their institutions are best prepared for demographic changes.

Through joint trainings over the course of the project, the exchange between colleagues in inpatient and outpatient settings can improve so that it is not only various occupational groups that benefit but also patients and family members. Due to the broad participation in the project, it is already becoming apparent that the participating institutions have implemented numerous improvements to the care of geriatric patients.

### Side-Effects of Training Evaluation

The project has already contributed to important innovations for both the member organizations of the Quality Network of Geriatric Medicine and also for the educational work for health care of elderly, multimorbid patients, which are as follows:

- The educational measures and their evaluation include various occupational groups, hierarchy and management levels, as well as sectors in inpatient and outpatient settings.
- Unlike the usual initiatives, the evaluation not only includes conducting the course but also the initial conditions as well as the implementation of learnings at the institutions.
- The evaluation includes gaining knowledge and educational success, but it also focuses on relevant outcome variables to identify treatment improvements for elderly patients and their family members. New methods are developed and tested for this purpose.
- The evaluation is itself part of the change and improvement process in which adaptive questioning and testing instruments are used, which contribute to the various improvements in the institutions. It remains to be seen whether the methods developed in the project such as the evaluation surveys and the process model for the consensus groups will be adjusted by the institutions for their purposes and adopted.
- The evaluation of various measures in 1 network is the first of its kind. This comprehensive evaluation can provide stimulus for the entire interconnected health care sector.

The overall links between training needs, training assessment, implementation efforts, and measurable results can only be assessed once the project has finished. The findings will be considered in the development and implementation of later professional educational work and its evaluation. The interprofessional educational initiative not only consists of various training courses but also their evaluation. One important element is the exchange between professionals, which also acts as promotion for the initiative itself. The competitive edge, which results from the professional range of treatment and care options in the Quality Network of Geriatric Medicine in north-west Germany, is not to be underestimated. Upon completion of the project, the full results will be made available to the public so that other institutions can also benefit from the results.

## Abbreviations

SF: Short Form
